# Modulation of dendritic cell functions by viral IL-10 encoded by human cytomegalovirus

**DOI:** 10.3389/fmicb.2014.00337

**Published:** 2014-07-04

**Authors:** Selmir Avdic, Brian P. McSharry, Barry Slobedman

**Affiliations:** ^1^Human Cytomegalovirus Research Group, Discipline of Infectious Diseases and Immunology, University of SydneyCamperdown, NSW, Australia; ^2^Centre for Virus Research, Westmead Millennium InstituteWestmead, NSW, Australia

**Keywords:** dendritic cells, cytomegalovirus, interleukin 10, immunomodulation, virus replication and latency

## Abstract

Human cytomegalovirus (HCMV), a clinically important β-herpesvirus, is a master of evasion and modulation of the host immune system, including inhibition of a number of dendritic cell (DC) functions. DCs play a central role in co-ordination of the immune response against pathogens and any disturbance of DCs functions can result in a cascade effect on a range of immune cells. Recently, the HCMV gene *UL111A*, which encodes viral homologs of human interleukin 10, has been identified as a strong suppressor of a number of DCs functions. In this mini review, we focus on HCMV-encoded viral IL-10-mediated inhibitory effects on DCs and implications for the development of an effective HCMV vaccine.

## HUMAN CYTOMEGALOVIRUS

Human cytomegalovirus (HCMV) is a species-specific β-herpesvirus that infects a majority of the world’s population. Primary HCMV infection in the immunocompetent host is generally asymptomatic, with a minority of persons experiencing HCMV induced mononucleosis. However, in immunocompromised patients, such as allogeneic stem cell and solid organ transplant recipients and HIV/AIDS patients, HCMV infection results in severe disease affecting multiple organs that can be fatal (Mocarski et al., 2013). Furthermore, HCMV is the most common viral congenital infection which can lead to permanent disability such as hearing loss, mental retardation, and neurological defects (Kenneson and Cannon, 2007).

Following primary infection, HCMV establishes a life-long latent infection within myeloid progenitor cells. These precursor cells can then differentiate into a number of immune cell types including, monocytes, macrophages and dendritic cells (DCs), that play a vital role in functions such as antigen presentation leading to pathogen control and clearance (Reeves and Sinclair, 2008). During latency the virus persists without detectable replication; however, HCMV periodically reactivates from latent infection, resulting in production and shedding of infectious virus, and this process is most clinically important in immunosuppressed patients, particularly in allogeneic stem cell and solid organ transplant recipients, in which reactivating virus is the major cause of serious disease.

Human cytomegalovirus consists of a large, double-stranded DNA genome that encodes a range of immunomodulatory genes which function to limit both innate and adaptive host immunity. Such functions are likely to contribute to the lifelong persistence that follows primary infection that is characteristic of HCMV infection. In particular, HCMV encodes a number of soluble molecules that are homologs of host encoded cytokines and chemokines (McSharry et al., 2012). The best characterized of these proteins is the viral homolog of human IL-10 for which a range of immunomodulatory functions have been identified, and is the focus of this review.

## HCMV-ENCODED HOMOLOGS OF HUMAN INTERLEUKIN 10

The human cytokine interleukin 10 (hIL-10) is a master regulator of the immune response, controlling functions of a number of different immune cell types (Moore et al., 2001). hIL-10 has particularly potent and varied effects on DCs. DC exposure to hIL-10 reduces their ability to activate and maintain immune responses, marked by suppression of pro-inflammatory cytokine production, expression of MHC class II and co-stimulatory molecules, DC maturation and ability to stimulate T cells (Hawrylowicz and O’Garra, 2005). The importance of IL-10 (either host cellular IL-10 or viral homologs of IL-10) in modulating DC function and controlling the anti-viral immune response is exemplified by the range of viruses infecting humans that either induce hIL-10 (e.g., HIV and hepatitis C virus; Reiser et al., 1997; Brockman et al., 2009) or encode their own homologs of this cytokine (e.g., Epstein Barr Virus and some *cytomegaloviruses*; Slobedman et al., 2009).

The HCMV gene *UL111A* encodes two viral homologs of human interleukin 10 (hIL-10); cmvIL-10 and LAcmvIL-10 that are both splice variants of the UL111A transcript. cmvIL-10 was identified first (Kotenko et al., 2000; Lockridge et al., 2000). This homolog shares only 27% amino acid identity with hIL-10, however, despite low homology with hIL-10, cmvIL-10 binds strongly and can signal through the hIL-10 receptor (Kotenko et al., 2000; Jones et al., 2002). LAcmvIL-10 is the second viral homolog of hIL-10 encoded by *UL111A* that has a truncated C-terminus due to alternative splicing (Jenkins et al., 2004), and together with cmvIL-10 makes up HCMV-encoded viral IL-10. Through intensive research activities of a number of groups over the last decade, a significant amount of data has been published demonstrating strong immunomodulatory functions of these viral IL-10 homologs on a range of human immune cells including DCs, monocytes, macrophages, and B cells (Spencer et al., 2002, 2008; Chang et al., 2004; Raftery et al., 2004; Yamamoto-Tabata et al., 2004; Jenkins et al., 2008; Cheung et al., 2009; Jaworowski et al., 2009; Slobedman et al., 2009; Avdic et al., 2011, 2013; McSharry et al., 2012). This review will principally focus on the effects of HCMV-encoded viral IL-10 on the function of DCs; cells often referred to as the sentinels of immunity, due to their exceptional ability to stimulate the immune response against pathogens.

## DC MODULATION BY HCMV-encoded HOMOLOGS OF hIL-10

Dendritic cells play a central role in linking and co-ordination of the innate and adaptive immune responses against foreign antigens. The DC family can be broadly divided into classical DCs (cDCs) which are comprised of Langerhans cells (LC), myeloid DCs and plasmacytoid DCs (pDCs; Caux et al., 1996; Grouard et al., 1997; Merad et al., 2013). Upon antigen encounter, immature cDCs undergo the maturation process, where they migrate to lymph nodes, process and present antigens via MHC class I and class II to prime CD8^+^ and CD4^+^ T lymphocytes, and also produce cytokines that further orchestrate an effective host immune response (De Smedt et al., 1996; Merad et al., 2013). Due to technological advances in flow cytometry and identification of novel cell surface markers over the last 10–15 years, new subsets of cDCs are continuing to be identified (Kim et al., 2014) and have been recently reviewed elsewhere (Merad et al., 2013). pDCs, on the other hand, represent a much smaller DC population that respond to initial viral stimuli primarily by strong production of the anti-viral cytokine, interferon alpha (IFN-α), but also retain the ability to prime T cells (Colonna et al., 2004).

Human cytomegalovirus infection has been shown to involve direct interaction with DCs at a number of levels. Firstly, HCMV has been demonstrated to infect both immature and mature DCs *in vitro* (Jahn et al., 1999; Riegler et al., 2000; Moutaftsi et al., 2002; Hertel et al., 2003). Secondly, HCMV can latently infect DC precursors (Hahn et al., 1998) and DCs have been identified as a site of HCMV reactivation from natural latent infection (Reeves et al., 2005; Reeves and Sinclair, 2013). It therefore comes as little surprise that HCMV has developed multiple strategies to inhibit a range of DC functions with an ultimate goal of limiting or preventing virus clearance by the immune system. While some aspects of this multi-targeted approach of DC modulation by HCMV have been characterized (e.g., disturbance of MHC class I and class II antigen presenting pathways by products of HCMV genes *US2-11*; Jones et al., 1996; Wiertz et al., 1996; Ahn et al., 1997; Machold et al., 1997; Tomazin et al., 1999), not all of HCMV-driven modulatory effects on DCs have been assigned to specific HCMV gene functions (Sinclair, 2008; Rolle and Olweus, 2009). However, it has become increasingly apparent that a number of DC functions are regulated by viral IL-10.

The first evidence of an HCMV-encoded IL-10 targeting DC function was reported by Peter Barry’s group. Utilizing supernatants harvested from either infections with a *UL111A* deletion mutant virus or the parental (viral IL-10-expressing) HCMV strain, they identified that HCMV-encoded viral IL-10 plays an important role in regulating DC maturation (Chang et al., 2004). Indeed, progression of immature monocyte derived DCs (MDDCs), representing an *in vitro* model of cDC (Sallusto and Lanzavecchia, 1994), into the mature phenotype induced by lipopolysaccharide (LPS) was prevented by culture supernatants from cells infected with parent virus but not from supernatants from cells infected with the *UL111A* deletion mutant. Inhibition of DC maturation by cmvIL-10 protein is accompanied by suppression of pro-inflammatory cytokine synthesis by recombinant cmvIL-10 protein, whereas the LAcmvIL-10 protein did not exert this effect on DCs (Chang et al., 2004; Jenkins et al., 2008). Similarly, cmvIL-10 (but not LAcmvIL-10) suppresses a range of MDDC surface co-stimulatory molecules: CD80, CD83, CD86, and CD40, which are required for efficient T cell activation (Raftery et al., 2004; Jenkins et al., 2008). These studies not only demonstrated profound suppression of DC maturation by HCMV-encoded IL-10, but also highlighted a divergence in the functions of cmvIL-10 and LAcmvIL-10, showing that LAcmvIL-10 retains some (e.g., suppression of cell surface MHC class II complex expression required for antigen presentation to CD4^+^ T cells) but not all functions of cmvIL-10 (Jenkins et al., 2008).

In addition to limiting the ability of cmvIL-10 treated cDCs to efficiently stimulate immune responses, exposure of mature cDCs to cmvIL-10 (and hIL-10) also results in rapid cell apoptosis via suppressing the induction of anti-apoptotic genes *bcl-2, bcl-x,* and *bfl-1* (Chang et al., 2007). Such effects are likely to limit the time-frame of antigen presentation and stimulation of T cells by cDCs exposed to cmvIL-10. A study by Raftery et al. (2004) similarly reported that cmvIL-10-induced cDC apoptosis in the presence of an inflammatory signal, which was linked to a reduction of anti-apoptotic c-FLIP_L_ mRNA expression. This comprehensive study also reported several other mechanisms of cmvIL-10-driven control of cDC functions, including partial suppression of MHC class I and class II as well as co-stimulatory molecules B7-H1 and B7-DC, ultimately resulting in suppressed ability of cmvIL-10-treated cDCs to induce proliferation of both allogeneic and autologous T cells (Raftery et al., 2004). Adding to this, cmvIL-10 also suppresses transcription of CD1 non-classical MHC class I molecules that are involved in presentation of lipids rather than peptides (Raftery et al., 2008).

Decreased cDC stimulatory activity is not due to suppression of antigen uptake by cmvIL-10-exposed cDCs. Rather, cmvIL-10 appears to actually stimulate antigen uptake in immature MDDCs (Raftery et al., 2004). This study also reported that surface expression of indoleamine 2,3-dioxygenase (IDO) was stimulated by cmvIL-10. IDO functions to suppress T cell responses by depletion of tryptophan (Hwu et al., 2000) and it has been suggested that IDO expressing cDCs may be involved in induction of T cell anergy (Munn et al., 2002). While it remains to be examined in an experimental setting, it is possible that increased antigen uptake by cmvIL-10-treated IDO expressing cDCs may lead to an increased proportion of HCMV antigens presented by IDO^+^ cDCs that could lead to an increased anergic role of anti-HCMV T cells.

The inhibitory effects of cmvIL-10 are not limited to cDC. cmvIL-10 released from HCMV-infected cells also effectively suppresses IFN-α mRNA transcription and protein synthesis by pDCs (Chang et al., 2009). This is biologically important in the context of HCMV infection as IFN-α stimulates other immune cells to clear the virus but also protects the same immune cells from HCMV infection (Chang et al., 2009).

Human cytomegalovirus establishes latent infection in CD34^+^ myeloid progenitors, which can differentiate into DCs or monocyte/macrophages. mRNA expression from the viral IL-10-encoding HCMV gene *UL111A* has been detected during experimental as well as natural HCMV latent infection (Jenkins et al., 2004; Cheung et al., 2006), and work using *UL111A* deletion viruses has shown that *UL111A* gene products suppress a number of immunostimulatory functions during HCMV latency. Firstly, surface MHC class II expression levels in myeloid progenitors latently infected with a parental, viral IL-10-expressing virus, are significantly lower than in progenitors infected with a *UL111A* deletion virus (Cheung et al., 2009). Consequently, stimulation of both allogeneic and autologous CD4^+^ T cell proliferation by progenitors carrying latent virus able to express viral IL-10 is notably suppressed compared to counterparts latently infected with the *UL111A* deletion virus (Cheung et al., 2009). Apart from their inability to prime CD4^+^ T cell responses, latently infected myeloid progenitors are also restricted by viral IL-10 in their ability to differentiate into myeloid DCs and LCs, with noted absence of pro-inflammatory cytokine expression by cells infected with the virus capable of expressing viral IL-10 (Avdic et al., 2011).

In addition to differentiation to DCs, myeloid progenitors can also differentiate into monocytes and macrophages which can act in antigen presenting roles. However, it appears that even these cells are impacted by viral IL-10, as exposure of these cells to viral IL-10 results in promotion of deactivated M2c monocytes/macrophages, rather than the classically activated, pro-inflammatory M1 type. This impact of viral IL-10 on monocyte/macrophage programming results in impairment of effective priming and proliferation of CD4^+^ T cells (Avdic et al., 2013).

Whilst viral IL-10 appears to minimize various DC functions required to instruct the immune system to clear the virus, this viral cytokine has also been reported to increase surface expression of DC-SIGN on immature MDDCs (Raftery et al., 2004). DC-SIGN is a lectin expressed on the cell surface of immature DCs and has been identified as a potential receptor for HCMV entry (Halary et al., 2002). Although it remains to be determined whether viral IL-10 plays any role in HCMV infection of DCs, it is possible that any viral IL-10-mediated upregulation of DC-SIGN may enhance virus entry into DCs.

## BLOCKING VIRAL IL-10: POTENTIAL HCMV VACCINE STRATEGY

The quest to develop a HCMV vaccine has been underway for >40 years (Elek and Stern, 1974). To this date, however, there is still no licensed HCMV vaccine. Multiple factors have complicated the development of an effective HCMV vaccine, with a significant limitation being the strict host specificity of HCMV, limiting the use of pre-clinical animal models to evaluate vaccine efficacy.

As already discussed, DCs are a critical component of the orchestrated immune response to pathogens that form a link between innate and adaptive immunity. For that reason, it is an imperative to ensure that DCs function at their best, instructing the immune system during initial response to vaccination and during subsequent challenge with live pathogens. Given that HCMV-encoded viral IL-10 severely alters DC functions, it could be argued that viral IL-10 expressed by any live attenuated HCMV vaccine candidate may limit the host’s immune response toward such a vaccine. Indeed, several recent studies have investigated the role of viral IL-10 in the development of a vaccine against HCMV. Due to strict species specificity of HCMV, these studies have relied upon a rhesus macaque animal model, whereby rhesus monkeys are infected with rhesus CMV (RhCMV), which also encodes a viral IL-10. It is important to note that murine cytomegalovirus (MCMV) does not encode its own IL-10 homolog and this has limited the study of cytomegalovirus encoded IL-10 in more common animal models.

Two approaches have been utilized to investigate immune responses when the expression of viral IL-10 is blocked or modified. Firstly, it has been demonstrated that the blockade of RhCMV-encoded viral IL-10 expression (using a viral IL-10 knockout virus) during RhCMV infection *in vivo* resulted in a significant increase in DC numbers in draining lymph nodes and stronger CD4^+^ T cell proliferation, as well as higher IgG titers (Chang and Barry, 2010). This study provided evidence that application of any live attenuated HCMV vaccine may be more effective at inducing anti-viral immunity if it contains targeted disruption of the viral IL-10 locus.

Secondly, it is known that antibodies are produced against viral IL-10 in both humans and rhesus macaques and that these can neutralize viral IL-10 function (de Lemos Rieper et al., 2011; Eberhardt et al., 2012). Therefore, blocking expression of viral IL-10 through gene deletion may limit a potential target of an effective immune response. An alternative approach to develop a vaccine whereby mutated versions of viral IL-10 unable to bind to the IL-10R but still able to stimulate production of anti-viral IL-10-neutralizing antibodies has also been investigated. Immunization of seronegative animals with mutated forms of viral IL-10 prior to challenge with RhCMV resulted in decreased viral replication both locally (at the site of RhCMV infection) and systemically (with reduced viral shedding in bodily fluids; Logsdon et al., 2011; Eberhardt et al., 2013).

Collectively, these data demonstrate that abrogating the immunosuppressive functions of viral IL-10, whilst still enabling generation of anti-viral IL-10 antibody, may be an important component in the design of an effective vaccine against HCMV.

## CONCLUSION

Viral IL-10 encoded by HCMV *UL111A* employs a multi-targeted approach to profoundly modulate DC functions. It decreases DC differentiation from progenitor cells and DC maturation from immature DCs. Suppression of antigen presenting molecules (MHC class I and class II) together with suppression of co-stimulatory molecules both work together to reduce priming and proliferation of T cells. Additionally, viral IL-10 also suppresses secretion of pro-inflammatory cytokines, which is likely to add to repression of the immune response. Viral IL-10 can reduce the ability of DCs to present antigen via inhibition of anti-apoptotic factors but also potentially drives DCs toward a phenotype that could induce T cell anergy and therefore render the adaptive immune response less responsive to the virus infection. Together with other immunommodulatory genes encoded by HCMV, the impact of viral IL-10 on DCs is likely to create an environment that benefits the virus by limiting virus clearance by the host immune system.

## Conflict of Interest Statement

The authors declare that the research was conducted in the absence of any commercial or financial relationships that could be construed as a potential conflict of interest.
